# β-Sitosterol 3-*O*-D-glucoside increases ceramide levels in the stratum corneum via the up-regulated expression of ceramide synthase-3 and glucosylceramide synthase in a reconstructed human epidermal keratinization model

**DOI:** 10.1371/journal.pone.0248150

**Published:** 2021-03-08

**Authors:** Shogo Takeda, Shuko Terazawa, Hiroshi Shimoda, Genji Imokawa

**Affiliations:** 1 Research & Development Division, Oryza Oil & Fat Chemical Co., Ltd., Ichinomiya, Aichi, Japan; 2 Center for Bioscience Research and Education, Utsunomiya University, Utsunomiya, Tochigi, Japan; Xiangtan University, CHINA

## Abstract

β-Sitosterol 3-*O*-d-glucoside (BSG) is known to act as an agonist by binding to estrogen receptors, and estrogen has been reported to enhance the activity of β-glucocerebrosidase, an epidermal ceramide metabolizing enzyme. In this study, we determined whether BSG up-regulates ceramide levels in the stratum corneum (SC) of a reconstructed human epidermal keratinization (RHEK) model. Treatment with BSG significantly increased the total ceramide content by 1.2-fold compared to that in the control in the SC of the RHEK model, accompanied by a significant increase of the ceramide species, Cer[EOS] by 2.1-fold compared to that in the control. RT-PCR analysis demonstrated that BSG significantly up-regulated the mRNA expression levels of serine palmitoyltransferase (SPT)2, ceramide synthase (CerS)3, glucosylceramide synthase (GCS) and acid sphingomyelinase by 1.41–1.89, 1.35–1.44, 1.19 and 2.06-fold, respectively, compared to that in the control in the RHEK model. Meanwhile, BSG significantly down-regulated the mRNA expression levels of sphingomyelin synthase (SMS)2 by 0.87–0.89-fold. RT-PCR analysis also demonstrated that BSG significantly up-regulated the mRNA expression levels of CerS3 and GCS by 1.19–1.55 and 1.20-fold, respectively, but not of SPT2 and significantly down-regulated that of SMS2 by 0.74-fold in HaCaT keratinocytes. Western blotting analysis revealed that BSG significantly increased the protein expression levels of CerS3 and GCS by 1.78 and 1.28–1.32-fold, respectively, compared to that in the control in HaCaT cells. These findings indicate that BSG stimulates ceramide synthesis via the up-regulated expression levels of CerS3 and GCS in the glucosylceramide pathway, which results in a significantly increased level of total ceramides in the SC accompanied by significantly increased levels of acylceramide species such as Cer[EOS].

## Introduction

Ceramides are comprised of sphingolipids consisting of a sphingoid base and a saturated fatty acid moiety. Ceramides are the dominant intercellular lipid in the stratum corneum (SC) and together with other lipids such as free fatty acids and cholesterol ester, they play essential roles as a water reservoir and as a barrier [1]. At this time, 12 major classes of ceramide profiles have been found in human SC [2]. Ceramides in the SC are produced via sequential enzymatic reactions of several sphingolipid metabolizing enzymes such as serine palmitoyltransferase (SPT), ceramide synthase (CerS), glucosylceramide synthase (GCS), sphingomyelin synthases (SMS), β-glucocerebrosidase (GBA) and acid sphingomyelinase (ASM). SPT catalyzes the condensation of serine and palmitoyl-CoA as the first step of *de novo* sphingolipid synthesis [3] and CerS catalyzes the synthesis of the basic ceramide structure by the *N*-acyltranslation of fatty acids [4]. GCS plays a role in glucosylceramide synthesis [5] and SMS inserts phosphorylcholine into the intermediate ceramide to yield sphingomyelin [6]. Ceramides in the SC are finally generated following the hydrolysis of sphingomyelin and glucosylceramide by ASM and GBA, respectively, in the interface between the SC and the stratum granulosum [7, 8]. Ceramides are subsequently hydrolyzed by acid ceramidase to yield sphingosine, the deficiency of which is predominantly associated with the *Staphylococcus aureus* colonization frequently observed in the SC of patients with atopic dermatitis (AD) [9].

Ceramides in the SC are significantly diminished in several dry skin diseases such as xerosis [10] and AD [11], generally accompanied by a water deficiency and/or barrier disruption in the SC. Even in healthy human skin, ceramide deficiencies can also occur with increasing age, being a prerequisite factor for age-related dry skin [11]. Recently, sensitive skin has been implicated to be derived from the down-regulated levels of ceramides in the SC that elicit a predisposition toward sensitive skin via a slight permeability barrier disruption in the SC [12]. Thus, dry skin symptoms have become a serious problem around the world and have prompted us to develop skin moisturizers with a potent ability to prevent or improve the integrity of dry skin by compensating for the ceramide loss in the SC via topical application of natural or synthetic ceramides and/or by stimulating ceramide synthesis in the epidermis via topical or oral treatments. Available studies have demonstrated that the oral administration of sphingolipids such as glucosylceramide and sphingomyelin improve skin barrier and water reservoir functions [13–15], although increased ceramide levels in the SC have not yet been demonstrated. We have already established a novel method by which stimulatory effects on ceramide levels in the SC can be precisely evaluated in a reconstructed human epidermal keratinization (RHEK) model. Using that method, candidate chemicals or materials with the potential to stimulate the expression of ceramide metabolic enzymes are incubated in the RHEK model for 7 days prior to the development of the SC. The newly formed SC is then subjected to ceramide analysis following which ceramide levels in the SC can be quantitatively expressed as μg/mg SC protein [16]. Using this RHEK model to characterize the disrupted barrier and water reservoir mechanisms in AD skin, we have already found clinically important evidence that Th1 cytokines accentuate but Th2 cytokines attenuate the level of ceramides in the SC [17]. Th1 cytokines such as IFN-γ and TNF-α elicit a distinct increase of total SC ceramide levels by up-regulating the expression of GBA and/or ASM [17]. Some chemicals, such as sphingosylphosphorylcholine and retinoic acid, have been implicated to have the potential to increase total SC ceramide levels, and retinoic acid has been shown to down-regulate the expression of acid ceramidase [16]. As an example of a material with an ability to stimulate ceramide synthesis in this RHEK model, we recently demonstrated that a strawberry seed extract significantly increases the level of total SC ceramides in concert with increased levels of Cer[NS/NDS] via the stimulation of gene and/or protein expression of SPT, CerS3, GCS and GBA [18].

It is well known that post-menopausal women are physiologically characterized by the frequent appearance of dry skin compared with younger women. Consistent with that, Wu et al. [19] reported that levels of ceramides in the SC but not natural moisturizing factors such as amino acids are significantly down-regulated especially in post-menopausal women compared with younger women. Since estrogen has been implicated to enhance GCS and GBA activities in skin explants from fetal rats [20], estrogen probably plays a distinct role in increasing or maintaining the levels of SC ceramides in the skin of younger women. Thus, it seems reasonable to assume that an estrogen deficiency is associated at least in part with the frequently induced dry skin in post-menopausal women due to a ceramide deficiency.

Therefore, we thought it likely that the estrogen-like stimulation of human keratinocytes could be an effective factor to improve the levels of ceramides in the SC via their increased synthesis. In this connection, several recent reports have demonstrated that β-sitosterol 3-*O*-β-d-glucoside (BSG) and its aglycon, β-sitosterol, can act as agonists by binding to the estrogen receptor [21–25]. Thus, we hypothesized that BSG would be an effective candidate to stimulate ceramide synthesis in the epidermis via the activation of an estrogen signaling cascade that may be associated with the enhanced gene expression of sphingolipid metabolizing enzymes. BSG is a phytosterol glycoside that is found in various plants [26–29] and has also been reported to exhibit several bioactivities such as anti-diabetic [30], anti-tumor [31, 32] and analgesic [33] effects.

In this study, we evaluated the effects of BSG on ceramide levels in the SC and the expression levels of sphingolipid metabolic enzymes related to ceramide synthesis in a RHEK model and in immortalized HaCaT human keratinocytes. We show for the first time that BSG stimulates ceramide synthesis via the up-regulated expression levels of CerS3 and GCS in the glucosylceramide pathway, which results in significantly increased levels of total ceramides in the SC accompanied by significantly increased levels of acylceramide species such as Cer[EOS].

## Materials and methods

### Materials

HaCaT keratinocytes were kindly supplied by Kyushu University (Fukuoka, Japan). Dulbecco’s modified Eagle medium (DMEM) phosphate buffered saline (PBS) buffer, skim milk and trypsin (2.5 mg/mL) /EDTA (0.25 mg/mL) aqueous solution were purchased from Fujifilm Wako Pure Chemical Co. Ltd. (Osaka, Japan) and fetal bovine serum (FBS) was obtained from Biosera (Boussens, France). LabCyte EPI-MODEL and assay medium supplied by Japan Tissue Engineering Co., Ltd. (Aichi, Japan) were obtained for the RHEK model. TLC plates for high performance thin-layer chromatography (HPTLC) were obtained from Merck Millipore (Darmstadt, Germany). Cer [NS] and [AS] for ceramide standards were purchased from Matreya LLC (State College, PA, USA). Random primer and dNTP mixture were purchased from Invitrogen (California, USA). TB Green^®^ Premix DimerEraser, NucleoSpin^®^ RNA and PrimeScript^TM^ Reverse Transcriptase were purchased from Takara Bio Inc. (Kusatsu, Japan). Radio immunoprecipitation assay (RIPA) buffer, BCA protein assay kit, protease phosphatase inhibitor cocktail, and ECL Plus western blotting substrate were obtained from Thermo Fisher Scientific Inc. (Massachusetts, USA). Super Signal Can get signal solutions 1 and 2 were purchased from Toyobo Co., Ltd. (Osaka, Japan). Antibodies to serine palmitoyltransferase (SPT)2 (ab23696) and ceramide synthase3 (CerS)3 (ab28637) were obtained from Abcam, Inc. (Cambridge, UK). Antibodies to glucosylceramide synthase (GCS) (sc-50511) and sphingomyelin synthase (SMS)2 (sc-34048) were obtained from Santa Cruz Biotechnology, Inc. (Colorado, USA). The antibody to β-actin (A1978) was purchased from Sigma-Aldrich Co., LLC. (Massachusetts, USA). Horseradish peroxidase (HRP) conjugated goat anti-mouse IgG and HRP conjugated goat anti-rabbit IgG were obtained from Merck Millipore (Darmstadt, Germany).

### Preparation of BSG

**Fig 1:**
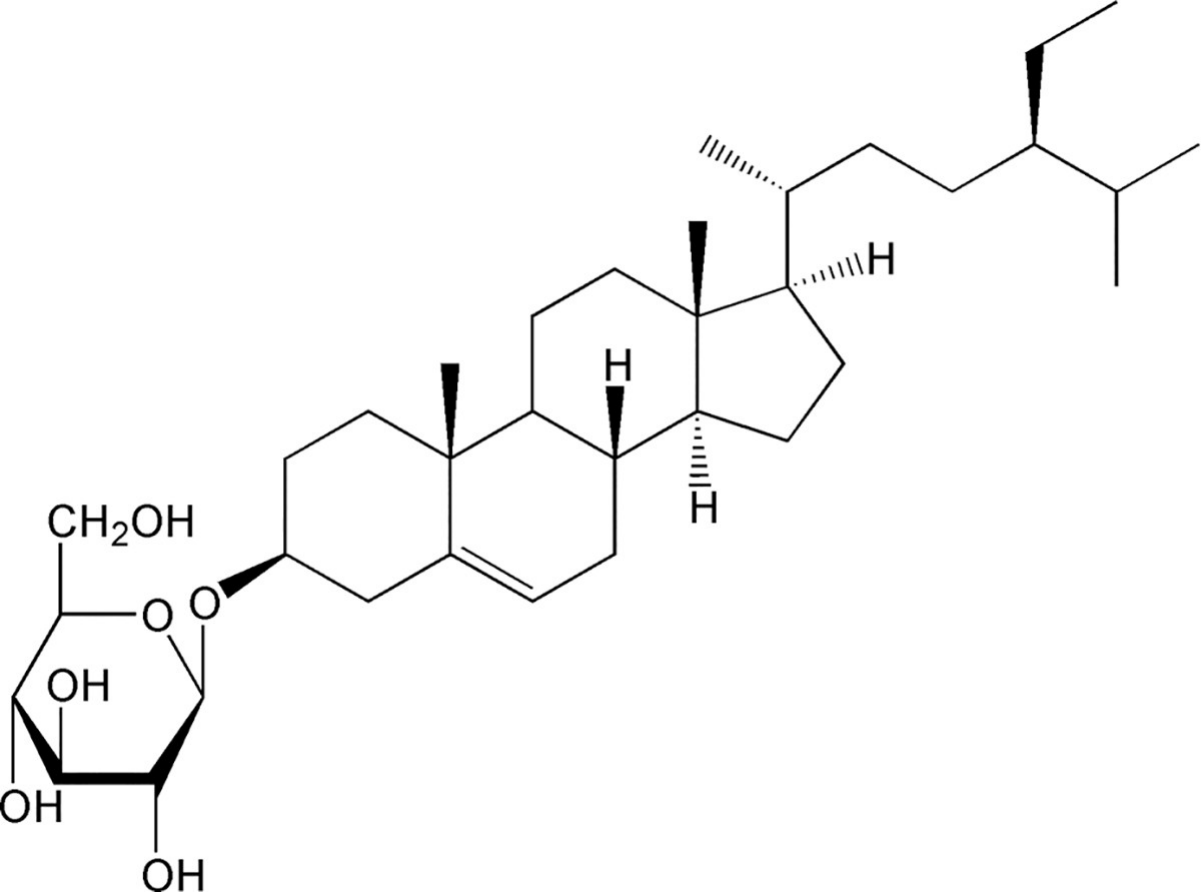
Chemical structure of BSG.

BSG was purified from a rice-derived glucosylceramide-rich fraction (GCF) manufactured from rice gum by Oryza Oil & Fat Chemical Co., Ltd. (Aichi, Japan). GCF was subjected to HPLC to isolate BSG. HPLC was performed under the following conditions using a refractive index detector (RID-10A; Shimazu, Kyoto, Japan). A silica gel column (TSK-GEL Silica-60, 20ϕ × 250 mm; Tosoh, Tokyo, Japan) and a C30 column (Develosil C30-UG-5; Nomura Chemical Co., Ltd., Aichi, Japan) were connected in tandem, and a mixture of chloroform, methanol and water (99:11:1) was used as a mobile phase. BSG was isolated as white amorphous powder and its structure was determined by comparing the ^1^H- and ^13^C-NMR spectra with the reported values [34]. The chemical structure of BSG is shown in Fig 1.

### RHEK model

RHEK models obtained prior to keratinization without development of the SC [35, 36] were used for quantification of ceramides and the expression of ceramide synthesis related enzymes. RHEK models consist of multi-layered human epidermal keratinocytes which is derived from neonatal foreskin and pre-cultured for 6 days prior to onset of our experiments. Each epidermal sheet of RHEK model was placed into 12- or 24-well culture plates for lipids analysis and real time RT-PCR, respectively, and assay medium was placed under the epidermal sheet [37]. DMEM/Ham’s F-12 (3:1) with 5% FBS was used as the assay medium. The assay medium added can be defused to basal layer of the RHEK model thorough the 0.4 μm pore of membrane. After incubation at 37°C in a 5% CO_2_ atmosphere for 1 day, the RHEK models were treated with BSG at final concentrations of 1, 3 and 10 μg/mL for culture times adjusted according to each experiment. Namely, the RHEK models were cultured for 2 or 4 days for analysis by real-time RT-PCR, and for 5 days for lipid analysis.

### Culture of HaCaT keratinocytes

HaCaT human keratinocytes were seeded at 4×10^4^ cells/well in 12-well culture plates and were maintained in DMEM with 10% FBS at 37°C in a 5% CO_2_ atmosphere. After overnight incubation, the medium was exchanged to DMEM without FBS and was incubated further for 24 hr, after which the cells were treated with BSG at final concentrations of 1, 3 and 10 μg/mL. The culture period was adjusted to 1, 3 or 6 hr for evaluation by real-time RT-PCR and to 48 hr for evaluation by western blotting analysis.

### Lipid extraction

The entire tissue of each RHEK model was removed from the membrane and was floated on trypsin (2.5 mg/mL) /EDTA (0.25 mg/mL) aqueous solution for 15 min at 37°C in a 5% CO_2_ atmosphere for the SC separation. After adding the FBS, the SC separation was then performed using a microscope. The Obtained SC were washed with PBS and stored at -80°C until the determination of ceramides. Bligh-Dyer method described in previous studies [17, 38] was used for lipid extraction. Briefly, homogenization of SC was carried out using an ultrasonic homogenizer (AGC Techno Glass Co., Ltd. Shizuoka, Japan) in a mixture of chloroform, methanol and PBS (1:2:0.8). The mixture was then centrifuged (840×g, 15 min) after which the supernatants were obtained. Subsequently, the same volume of chloroform and PBS were added to each supernatant, solution were stirred using a vortex mixer for 20 min. After stirring, the solution was centrifuged (840×g, 15 min) and the bottom layer was obtained using a glass syringe. The obtained layer was dried at 30°C by N_2_ gas. Remaining precipitates were used for quantification of total protein contents to correct the ceramide contents.

### Quantification of ceramides in the SC

Ceramide contents in the SC were measured by HPTLC. The quantification method described in a previous study [18] was carried out for TLC analysis. The lipid samples from SC were dissolved in a mixture of chloroform and methanol (2:1) for the TLC analysis. Lipid samples were developed using TLC plates (10×10 cm). Firstly, samples were developed by a mixture of chloroform, methanol and acetic acid (190:9:1) and a mixture of chloroform, methanol and acetic acid (197:2:1) were used as a second development. Developed plates were soaked with 10% copper sulfate in a 8% phosphoric acid aqueous solution and then heated at 180°C for 7 min for the spot visualization. Imaging system (ImageQuant LAS500; GE Health Care, Connecticut, USA) was used for the scanning and analysis of the ceramide spots. Each spot areas of ceramides were corrected for that of ceramide standards.

### Protein analysis of the SC

Protein contents in the SC of the RHEK models were used for correction of ceramide contents. Precipitates remaining after lipid extraction were dissolved in a mixture of 10% sodium dodecyl sulfate (SDS) and 1N NaOH (1:9) at 60°C for 2 h. The mixtures were then neutralized with 2N HCl and the total protein amounts were determined by the BCA method.

### Real time RT-PCR

mRNA expression levels of ceramide synthesis related enzymes were measured by quantitative real time RT-PCR. NucleoSpin^®^ RNA was used for the total RNAs extraction from whole RHEK models or from HaCaT cells. PrimeScript^TM^ Reverse Transcriptase was used for the reverse-transcription of 0.1 μg each total RNA. Real-time RT-PCR reactions were performed using TB Green^®^ Premix DimerEraser and Thermal Cycler Dice^®^ Real Time System Single (Takara Bio Inc., TM 800). Primers information are shown in Table 1. The mRNA expression level of gryceraldehyde-3-phpsphate dehydrogenase (GAPDH) was used to correct that of each enzymes.

**Table 1:** RT-PCR primer sequences.

Genes	Forward primer (5’-3’)	Reverse primer (5’-3’)
SPT2	AGCCGCCAAAGTCCTTGAG	CTTGTCCAGGTTTCCAATTTCC
CerS3	CCAGGCTGAAGAAATTCCAG	AACGCAATTCCAGCAACAGT
GCS	ATGTGTCATTGCCTGGCATG	CCAGGCGACTGCATAATCAAG
GBA	TGGCATTGCTGTACATTGG	CGTTCTTCTGACTGGCAACC
SMS2	AAGTGTATAACATCAGCTGTGAA	CAGTACCAGTTGTGCTAGACTAC
ASM	TGGCTCTATGAAGCGATGG	AGGCCGATGTAGGTAGTTGC
GAPDH	AAGGTGAAGGTCGGAGTCAAC	GGGGTCATTGATGGCAACAATA

### Western blotting

HaCaT keratinocytes were harvested and homogenized in RIPA buffer containing a protease phosphatase inhibitor cocktail and EDTA (0.5 M). The solution was centrifuged (4°C, 21,130×g, 20 min) to obtain the supernatant. After adding sample loading buffer (62.5 mM Tris-HCl, 5% 2-mercaptoethanol, 2% SDS, 25% glycerol and 0.01% bromophenol blue) to supernatant which adjusted to 1.0 mg/mL protein content, mixture were heated at 95°C for 5 min. Electrophoresis was performed 10% SDS gels. Polyvinylidene difluoride membranes were used for transcription of separated proteins and 5% skim milk was used for the blocking. Primary antibodies were used as follows; SPT2 (1:1,000), GCS (1:200), CerS3 (1:1,000), SMS2 (1:200) and β-actin (1:10,000) Secondary antibodies were used as follows; HRP-conjugated goat anti-rabbit IgG (1:10,000) and HRP-conjugated goat anti-mouse IgG (1:10,000). ImageQuant LAS500 and ECL Plus western blotting substrate were used for the detection.

### Statistics

One-way analysis of variance (ANOVA) followed by Dunnett’s test was performed for the significance test. All data are presented as means ± standard deviation (SD) and differences with *p*<0.05 are considered significant.

## Results

### Effects of BSG on ceramide contents in the SC

**Fig 2:**
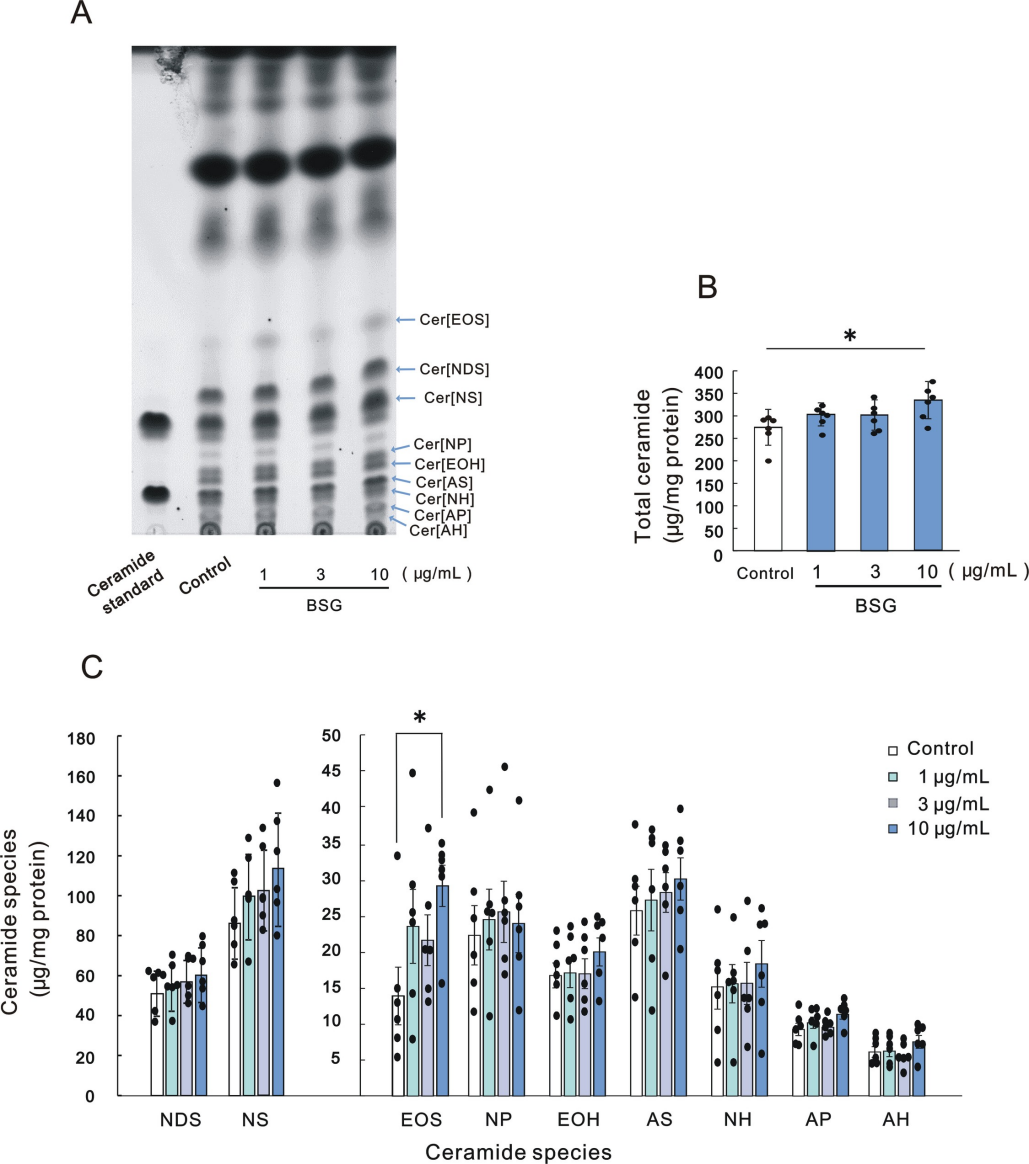
Effect of BSG on levels of ceramides in the SC of RHEK models. A: HPTLC chromatogram of lipids in the SC; B: Levels of total ceramides; C: Levels of ceramide species. RHEK models were treated for 5 days in culture without or with BSG (1, 3 and 10 μg/mL). The extraction of lipids from the SC of the RHEK model and HPTLC analysis were performed as described in Materials and methods section. Data are expressed as means ± SD (n = 6). *p<0.05.

Quantitative analysis of total ceramides and each ceramide species demonstrated that BSG at 10 μg/mL significantly increased the level of total ceramides in concert with the increased level of Cer[EOS] although the other ceramide species remained substantially unchanged (Fig 2B and 2C).

### Effects of BSG on mRNA levels of ceramide synthesis associated enzymes in RHEK models

**Fig 3:**
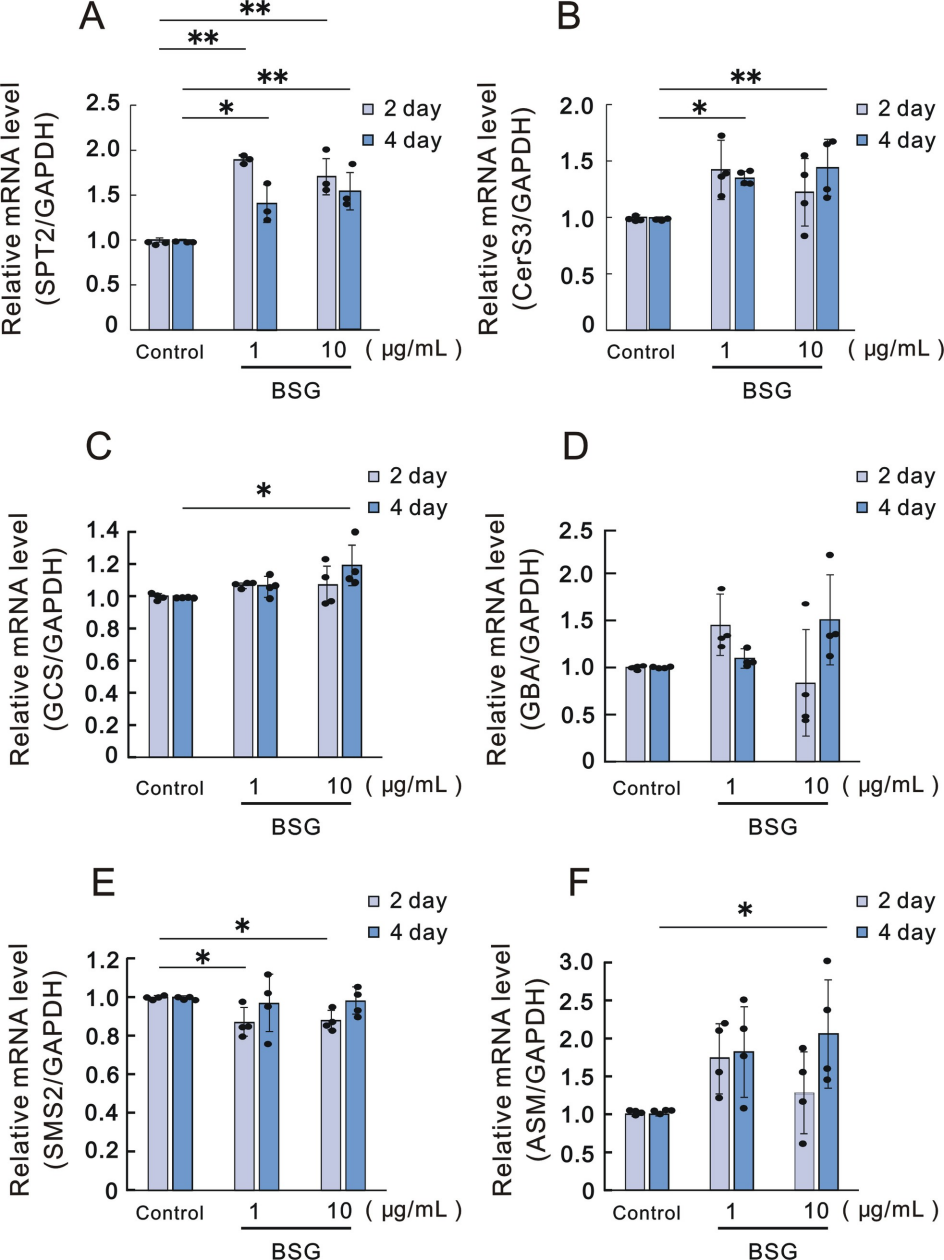
Effect of BSG on mRNA expression levels of enzymes involved in ceramide synthesis in RHEK models. A: SPT2, n = 3; B: CerS3, n = 4; C: GCS, n = 4; D: GBA, n = 4; E: SMS2, n = 4; and F: ASM, n = 4. RHEK models were cultured in the absence or presence of BSG at the indicated concentrations for the indicated number of days. Real time RT-PCR analysis was performed as described in the Materials and methods section. Data are expressed as means ± SD. *p<0.05, **p<0.01.

When RHEK models were treated with BSG for 2 and 4 days, the mRNA expression levels of SPT2, CerS3, GCS and ASM were significantly increased by BSG at 1 and 10, at 1 and 10, at 10 and at 10 μg/mL after 2 or 4, 4, 4 and 4 days of culture, respectively (Fig 3A, 3B, 3C and 3F). In contrast, the mRNA expression level of SMS2 was significantly decreased by BSG at 1 and 10 μg/mL after 2 days of culture (Fig 3E) while the mRNA expression level of GBA was not significantly changed (Fig 3D).

### Effects of BSG on mRNA expression levels of ceramide synthesis associated enzymes in HaCaT keratinocytes

**Fig 4:**
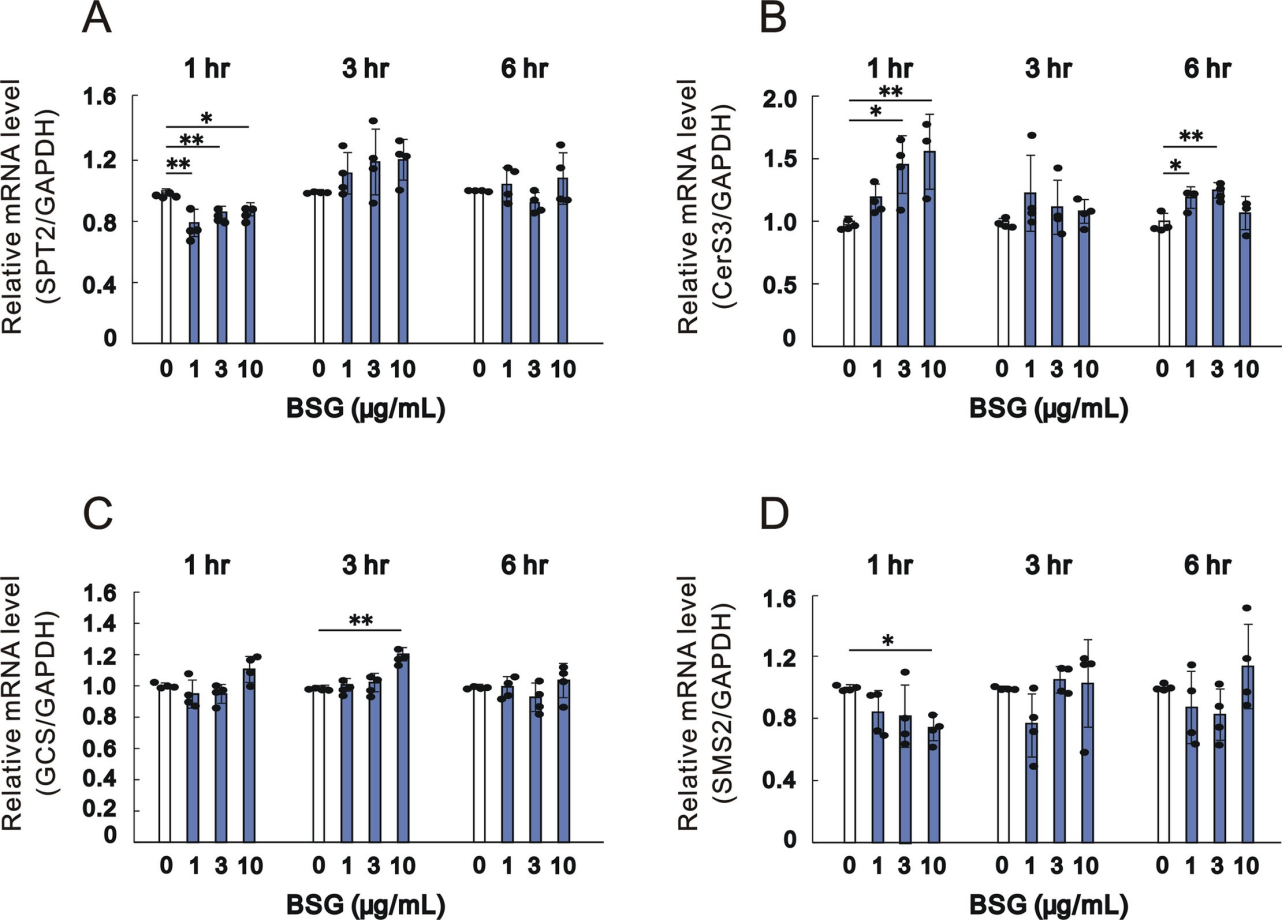
Effect of BSG on mRNA expression levels of enzymes involved in ceramide synthesis in HaCaT keratinocytes. A: SPT2, n = 4; B: CerS3, n = 4; C: GCS, n = 4; and D: SMS2, n = 4. HaCaT cells were cultured in the absence or presence of BSG at the indicated concentrations for the indicated number of hours. Real time RT-PCR analysis was performed as described in the Materials and methods section. Data are expressed as means ± SD from 4 independent experiments. *p<0.05, **p<0.01.

When HaCaT keratinocytes were cultured in the presence of BSG for 1, 3 and 6 hr, the mRNA expression levels of CerS3 and GCS were significantly increased by BSG at 3 and 10 and at 10 μg/mL after 1 and 3 hr of culture, respectively (Fig 4B and 4C). In contrast, the mRNA expression levels of SPT2 and SMS2 were significantly decreased by BSG at 1, 3 and 10 and at 10 μg/mL, respectively, only after 1 hr of culture (Fig 4A and 4D).

### Effects of BSG on protein expression levels of ceramide synthesis associated enzymes in HaCaT keratinocytes

**Fig 5:**
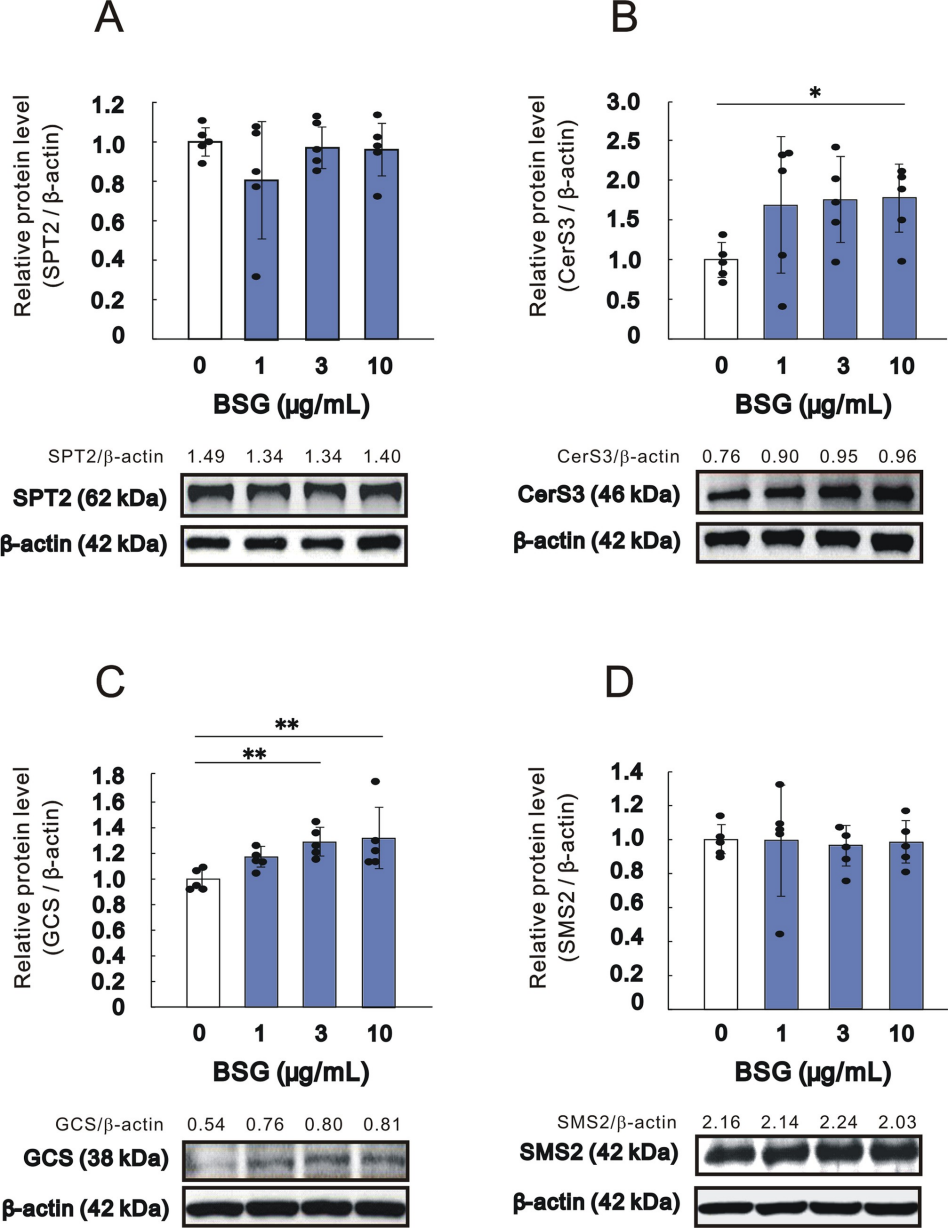
Effect of BSG on protein expression levels of enzymes involved in ceramide synthesis in HaCaT keratinocytes. A: SPT2, n = 5; B: CerS3, n = 5; C: GCS, n = 5; and D: SMS2, n = 5. HaCaT cells were cultured in the absence or presence of BSG at the indicated concentrations for 48 hr. Western blotting analysis was performed as described in the Materials and methods section. Representative immunoblots from 5 experiments are shown. Data are expressed as means ± SD from 5 independent experiments. *p<0.05, **p<0.01.

The protein expression levels of CerS3 and GCS were significantly increased by BSG at 10 and at 3 and 10 μg/mL, respectively, after 48 hr of culture (Fig 5B, 5C and S1B and S1C Fig). On the other hand, the protein expression levels of SPT2 and SMS2 remained unchanged by BSG at all concentrations tested (Fig 5A, 5D and S1A and S1D Fig).

## Discussion

As for the SC ceramide level, we found for the first time that BSG significantly increases the total SC ceramide level in the RHEK model in concert with a significantly increased level of acylceramide type Cer[EOS]. The acylceramide type ceramide has an esterified ω-hydroxy fatty acid group in the main chemical structure of ceramide and is well known to predominantly contribute to the barrier function of the SC [39, 40] via its riveting and stabilizing action for the ceramide constructed lamellar structure [41, 42]. Thus, BSG is a promising candidate compound that could repair aged skin with xerosis as well as barrier disrupted atopic dry skin.

There are two distinct ceramide synthetic pathways in the epidermis in which glucosylceramide and sphingomyelin serve as intermediate precursors [5]. Thus, while two-thirds of SC ceramide species including acylceramide are synthesized via the glucosylceramide pathway, the synthesis of the other one-third of SC ceramide species excluding acylceramide is mediated via the sphingomyelin pathway [43, 44]. As for the mechanisms by which BSG significantly increases the level of total ceramides in the SC, accompanied by the significantly increased level of the acylceramide type Cer[EOS], we found that BSG significantly up-regulates the mRNA expression levels of SPT2, CerS3, GCS and ASM in the RHEK model, whereas it significantly down-regulates the mRNA expression level of SMS2. A similar study utilizing HaCaT keratinocytes showed that BSG significantly enhances the mRNA expression levels of CerS3 and GCS, whereas the mRNA expression levels of SPT2 and SMS2 are significantly diminished. Western blotting analysis revealed that the protein levels of CerS3 and GCS are significantly increased by BSG treatment. Taken together, these findings suggest that BSG preferentially stimulates ceramide synthesis via the glucosylceramide pathway due to the up-regulated expression levels of CerS3 and GCS, which result in significantly accentuated levels of acylceramide synthesis such as Cer[EOS], leading in turn to the significantly increased level of total SC ceramides.

Several cytokines, chemicals and materials have been identified that have the potential to stimulate epidermal ceramide synthesis in the same RHEK model, resulting in the increased level of total ceramides in the SC. Those bioactive materials include Th1 cytokines such as IFN-γ and TNF-α, as well as sphingosylphosphorylcholine and retinoic acid whose stimulation of ceramide synthesis has been shown to be mainly mediated via the sphingomyelin pathway [16, 17]. We recently reported that a strawberry seed extract and its main constituent, tiliroside, significantly increases the total SC ceramide levels concomitant with the increased level of Cer[NS/NDS] via the up-regulated mRNA and/or protein expression levels of SPT, CerS3, GCS and GBA [18]. On the other hand, the present study demonstrated that BSG stimulates the glucosylceramide pathway by up-regulating the mRNA and protein expression levels of CerS3 and GCS, which results in the significant increase of total SC ceramides, accompanied by a significantly increased level of the acyl type ceramide, Cer[EOS].

As for the mechanisms by which the expression of CerS3 and GCS is significantly up-regulated in human keratinocytes of the RHEK model, it should be noted that phytosterols such as BSG are structurally similar to steroid hormones including estradiol and they potentially function as an estrogen [45, 46]. This estrogen-like action of BSG is corroborated by evidence that it can function as an estrogen-like compound with competitive binding to the estrogen receptor [22–25] to ameliorate glycogen synthesis in ovariectomized rats [21]. Available evidence indicates that estrogen has a potential to up-regulate the epidermal activities of GCS and GBA in skin explants from fetal rats [20]. Thus, it seems likely that the observed stimulatory effect of BSG on the expression levels of CerS3 and GCS in human keratinocytes results from the estrogen-like action of BSG although confirmation of that is under investigation.

**Fig 6:**
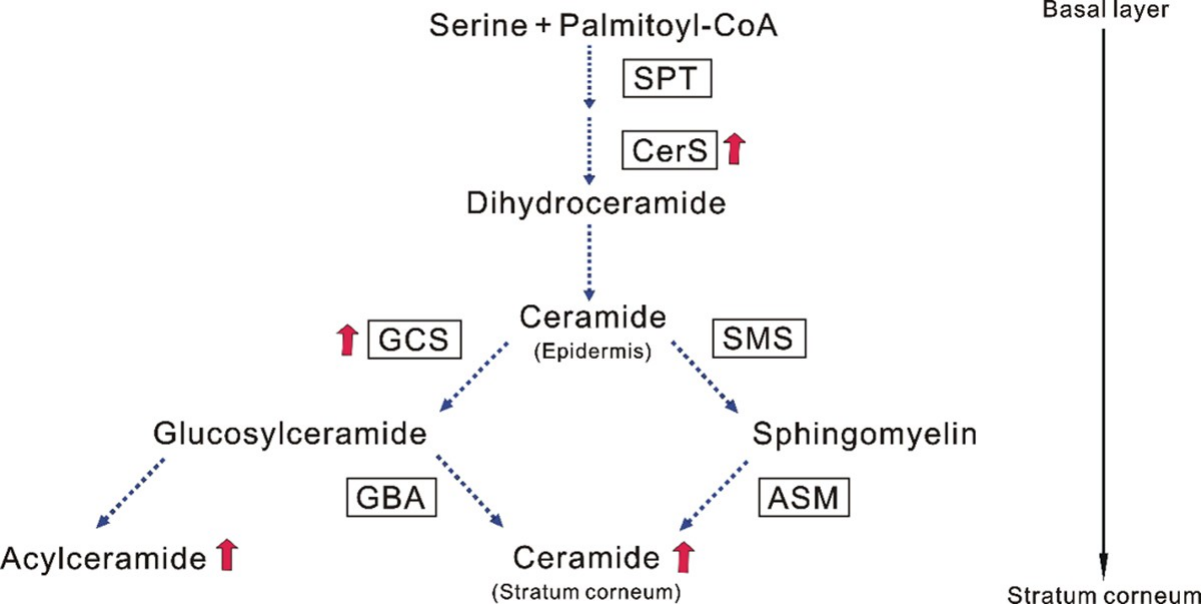
The mechanism of SC ceramide increasing effect of BSG. Increased levels of expression by BSG are indicated by red arrows.

In conclusion, as shown schematically in Fig 6, our results indicate that BSG has the potential to stimulate epidermal ceramide synthesis via the glucosylceramide pathway by enhancing the expression levels of ceramide metabolic enzymes, especially CerS3 and GCS. This results in significant increases in the levels of total ceramides in the SC in concert with the significantly up-regulated level of acylceramide type Cer[EOS]. Therefore, taken together, BSG is a promising bioactive compound with an ability to ameliorate aged dry skin including xerosis as well as to improve the barrier disrupted dry skin such as occurs in patients with AD.

## Supporting information

S1 Fig Supporting data for Fig 5. Data represent the original uncropped and unadjusted blots in Fig 5.
(TIF)

## Abbreviations

BSG: β-sitosterol 3-*O*-d-glucoside
SC: stratum corneum
RHEK: reconstructed human epidermal keratinization
Cer[EOS]: ceramide consisted with esterified-ωOH-fatty acid and sphingosine
Cer[NDS]: ceramide consisted with non OH-fatty acid and dihydrosphingosine
Cer[NS]: ceramide consisted with non OH-fatty acid and sphingosine
Cer[NP]: ceramide consisted with non OH-fatty acid and phytosphingosine
Cer[EOH]: ceramide consisted with esterified-ωOH-fatty acid and 6-hydroxysphingosine
Cer[AS]: ceramide consisted with αOH-fatty acid and sphingosine
Cer[NH]: ceramide consisted with non OH-fatty acid and 6-hydroxysphingosine
Cer[AP]: ceramide consisted with αOH-fatty acid and phytosphingosine
Cer[AH]: ceramide consisted with αOH-fatty acid and 6-hydroxyphytosphingosine
GAPDH: glyceraldehyde-3-phpsphate dehydrogenase
SPT: serine palmitoyltransferase
CerS: ceramide synthase
GCS: glucosylceramide synthase
GBA: β-glucocerebrosidase
SMS: sphingomyelin synthase
ASM: acid sphingomyelinase
AD: atopic dermatitis
PBS: phosphate buffered saline
RIPA: radioimmunoprecipitation assay
DMEM: Dulbecco’s modified Eagle medium
FBS: fetal bovine serum
HPTLC: high performance thin-layer chromatography
