# Supplementary figures and images for: β-Sitosterol 3-O-D-glucoside increases ceramide levels in the stratum corneum via the up-regulated expression of ceramide synthase-3 and glucosylceramide synthase in a reconstructed human epidermal keratinization model
Source: PLoS One. 2021 Mar 8;16(3):e0248150. doi: 10.1371/journal.pone.0248150 (PMC7939263; doi:10.1371/journal.pone.0248150)

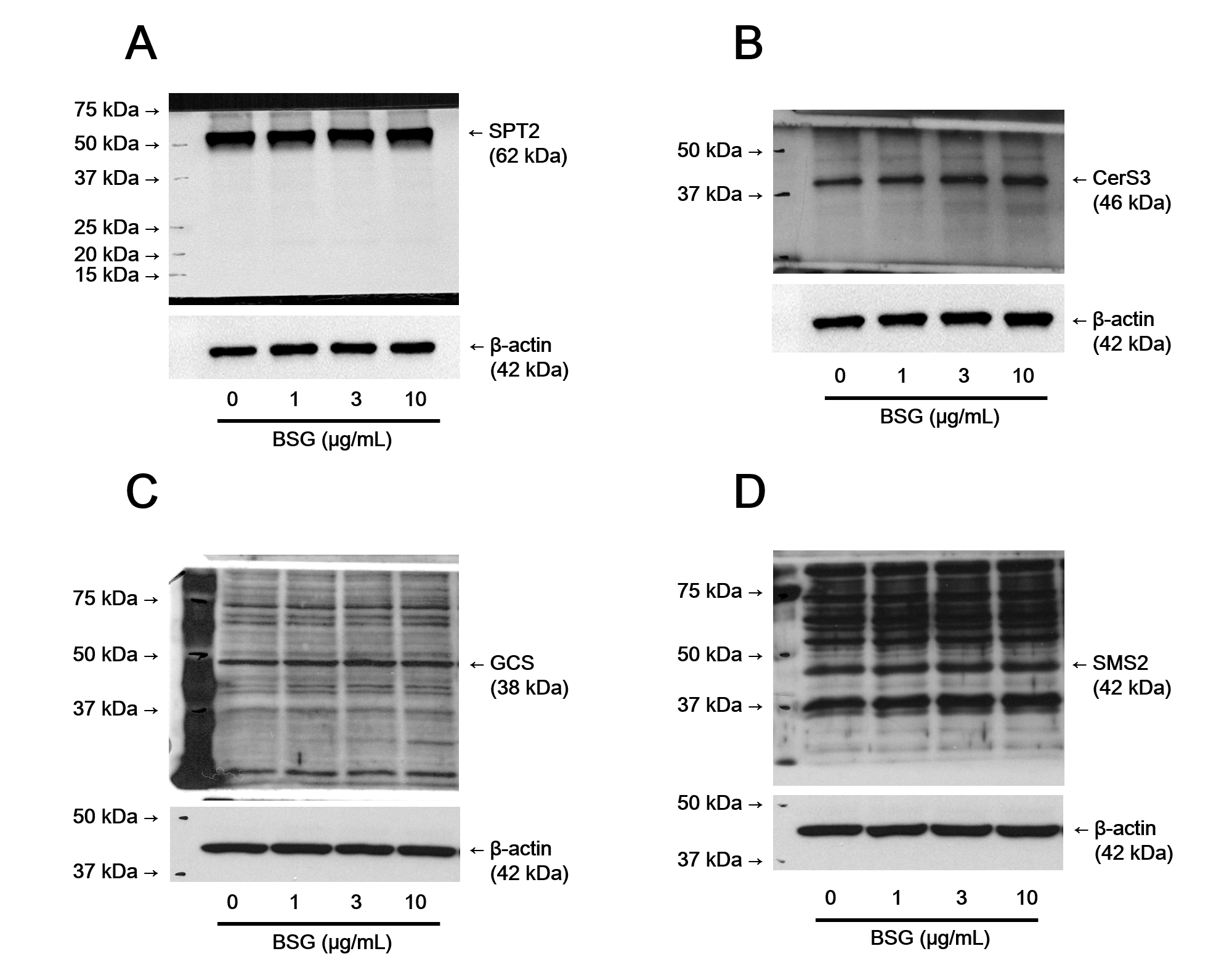

Supplement: S1 Fig — Data represent the original uncropped and unadjusted blots in Fig 5. (TIF) [file pone.0248150.s001.tif]
